# A note on statistical and biological communication: a case study of the 2009 H1N1 pandemic

**DOI:** 10.1186/1756-0500-7-939

**Published:** 2014-12-20

**Authors:** Jeremy Recoskie, Jane M Heffernan, Hanna K Jankowski

**Affiliations:** Department of Mathematics and Statistics, York University, Toronto, ON Canada; Centre for Disease Modelling, York Institute for Health Research, York University, Toronto, ON Canada

**Keywords:** Statistical misinterpretation, Miscommunication, Reproducibility, H1N1, Influenza

## Abstract

**Background:**

Many academic fields contribute to medical and health research. As a result, due to the various backgrounds of these disciplines, inference and interpretation of study findings can be misconstrued.

**Results:**

In a recent survey of the 2009 H1N1 literature we found many instances where semantic and statistical misinterpretation or miscommunication could potentially arise. We provide examples where miscommunication or misinterpretation of study results can mislead the interdisciplinary reader. We also provide some additional background on statistical methodology and theory for the interested reader.

**Discussion:**

This work presented some examples where statistical misinterpretation or miscommunication could arise in the H1N1 literature. However, similar challenges are encountered in other subjects and disciplines. To reduce the probability of this occurring it is necessary that (1) readers consider papers with a critical eye and approach citations with caution; (2) authors take more care to present study methods with more clarity. Reproducibility of the study results would greatly aid readers in their ability to understand and interpret the given findings.

## Introduction

Within the fields of medicine and health, there is a constant written dialogue through various medical journals, papers, and reports. Professionals within the academic disciplines of medicine, health, biology, statistics, and mathematics are primary contributors to these texts. Due to the complexity involving multiple disciplines, authors, and researchers, there is a clear need for a common language of dissemination so that the results of collaborative efforts may be more easily interpreted across fields.

In a recent survey of the 2009 H1N1 pandemic influenza literature, we came across several instances where semantic and statistical misinterpretation or miscommunication could potentially arise, and we give some of these examples here. The statistical examples we present include specific cases of important broad statistical concepts that are widely used in the biological and medical literature: including estimation, sample size considerations in hypothesis testing, and graphical methods. Although the examples are not exhaustive in covering the vast field of statistics, we believe that these examples could be of use to interdisciplinary research groups in biology, medicine and health, and researchers in mathematical biology.

### Organization of material

The examples are presented in the following sections, followed by a discussion. Remarks 1, 2, 3, 4 and 5 provide additional background on the statistical methodology and theory discussed in the main text, for the interested reader. A list of notations and abbreviations used in the main text is provided at the end of the note, immediately following the discussion.

## Representations and citations

With the vast amount of literature surrounding the study of influenza, challenges can arise in tracking results across publications, potentially leading to interpretations which differ from those intended by the original authors. We give some examples here, to illustrate possible misleading representations and citations. We focus on three studies by [1–3].

In the 2009 H1N1 pandemic, the effects of infection and vaccination in children were of some interest, and many studies included cohorts of children in their data. However, the definition of ‘child’ varied across some of these publications. For example, study cohorts ranged from 10 ‘infants’ ([1], mean age 7.6 months, 6.1–11.8 months age range) to a study of 124 children ([2, 3], ages 6 months to 9 years). It is important to note that, within these age ranges, immune system functions can vary considerably [4]. Thus, it is difficult to compare results over these ages and between these different cohorts. Therefore, the reader should interpret these results with some caution.

Citations within these papers also can appear somewhat misleading. For example, [1] cite [2] when writing “Middle-aged adults had been exposed repeatedly to seasonal influenza viruses, leading to antibody production, whereas young children often lacked previous exposures”. However, the result of [2] is “children had little evidence of cross-reactive antibodies to 2009 H1N1”, not that children lacked previous exposure to influenza leading to antibody production. Furthermore, [2] also concluded that “the data confirm the presence of some level of cross-reactive antibody in persons 60 years or more of age and the lack of such antibody in children and adults”.

Another example of a citation which could be misinterpreted also comes from [1], where it is stated that “young infants and children, as in previous pandemics, had high rates of infection with comparatively low mortality” and that “this paradox is explained by absence of protective and pathogenic immunity in children before infection”. Here, the authors are referring to CDC [3]. However, CDC [3] states that “the results indicated that before vaccination, no cross-reactive antibody to the novel influenza A (H1N1) virus existed among children”, as well as, “previous vaccination of children... did not elicit a cross-reactive antibody response to the novel influenza A (H1N1) virus”. Although the idea behind the statements from [1] and CDC [3] is the same, antibodies are only one form of immunity, and therefore it can be misleading for [1] to generalize such a statement when citing another study.

## Estimating a density and assessing goodness-of-fit

In data analysis, the complex behaviour of data can often be summarized through an appropriate choice of a statistical model. When researchers are interested in the distribution of some quantity, they often model this behaviour by fitting a probability density function to the data. Some popular choices of distributions used here include the normal, log-normal, or gamma densities. For an example, consider the incubation period of the H1N1 pandemic as estimated in [5]. The incubation period is defined as the time between infection of an individual and the appearance of symptoms. Here, the authors estimate the incubation period based on a sample size of *n* = 316 laboratory-confirmed cases of H1N1 and fit a log-normal distribution to the observed data. For further details on data acquisition and how missing data were handled we refer to [5].

In attempting to replicate the analysis of [5], we came across two specific issues with this data set: First, the data set has been discretized (we expect the true process to be continuous, whereas only integer values were observed). Second, the data set has several zero observations, whereas a log-normal density does not allow for observations of zero. Unfortunately, [5] do not discuss how they handle these issues. Here, we assume that an observation of 0 means that the incubation time was actually < 1, an observation of 1 means that the incubation time was larger than 1 but smaller than 2 (days), and so on. That is, we assume that the data have been *interval censored* or *grouped*. With this assumption, we use two popular approaches to estimate our model, maximum likelihood and least squares estimation.

First, we consider the method of maximum likelihood. To handle the discretized data we employ an approach very popular in insurance and actuarial applications: We assume that an observation known to fall somewhere in an interval (e.g. [1,2)) falls exactly at the midpoint of that interval (e.g. 1.5). In our case, it means that we transform the data as described in [5] by adding 0.5 to each integer value (i.e. an observation of 1 becomes 1.5, and observation of 0 becomes 0.5, etc.). Let us denote this transformed data as *z*_1_,…,*z*_316_. Now, we can find the maximum likelihood estimator of *θ* = (*μ*,*σ*), as described in Remark 1, when the density *f*(*x*|*θ*) is the log-normal density.

We next consider a least squares approach to estimate the unknown parameters. We handle the discretized data directly and without using the midpoint assumption as for maximum likelihood. The details of the method are described in Remark 2, with *θ* = (*μ*,*σ*) and log-normal density *f*(*x*|*θ*). The intervals, or bins, in our case were taken to be *I*_1_ = [ 0,1),*I*_2_ = [ 1,2),…,*I*_10_ = [ 9,10),*I*_11_ = [ 10,*∞*).

### Remark 1 (Maximum likelihood estimation (MLE) when data is not grouped)

Maximum likelihood is a popular statistical method used for parameter estimation. Let *f*(*x*|*θ*) denote the density model chosen and *θ*denote the unknown parameter(s) of the model. Let *z*_1_,…,*z*_*n*_ denote the observed data, where *n* denotes the sample size. The method of maximum likelihood says that the estimate of *θ* should be  the value of *θ* which maximizes the likelihood function


The estimated density model then becomes  Note that the above formula makes the implicit assumption that the observed data are independent.

### Remark 2 (Least squares estimation (LSE) when data is grouped).

The method of least squares is a second popular method to find the unknown parameter values in a model. As in Remark 1, let *f*(*x*|*θ*) denote the density model chosen and *θ* denote the unknown parameter(s) of the model. Assume that the data *z*_1_,…,*z*_*n*_ has been grouped (or binned) into *m* intervals *I*_1_,…,*I*_*m*_. Let  denote the proportion of observed data which lies in each bin. The method of least squares then says that the estimate of *θ* should be  the value of *θ* which *minimizes*

The estimated density model then becomes 

### Remark 3 (Goodness-of-fit)

Suppose the data are divided among *k* boxes: *B*_1_,…,*B*_*k*_. Now, calculate *O*_*i*_, the observed number of data in the *i*th box, and  the expected number of observations in the *i*th box under the estimated model. Here, *n* denotes the sample size. The larger the chi-squared test statistic


the more evidence we have that the model does not fit well. The *α*-critical value of the test statistics is the upper *α* quantile of the  distribution with *k* - *m* degrees of freedom, where *m* is the number of parameters estimated in the model. Equivalently, the *p*-value is calculated as  One “rule-of-thumb” states that the boxes should satisfy *E*_*i*_≥5, which improves the quality of the test.

**Figure 1:**
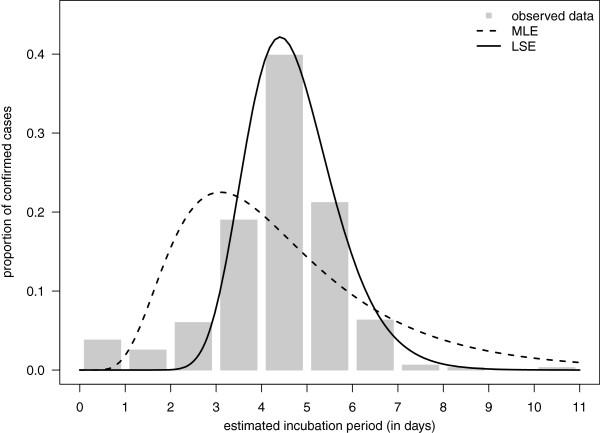
**Fitting the log-normal distribution to the incubation data.** Maximum likelihood and least squares estimators are fit to empirical incubation period data (see legend).

The results of both methods are shown in Figure 1, where we used fictional data similar to that given in ([5], Figure four). From Figure 1, it is evident that neither method appears to fit the overall data well. The LSE does a better job of modelling the main mode of the empirical data, whereas the MLE seems to fit the small values of the data better than the LSE, but does not capture the main mode of the observations. Neither method handles the small values well, especially the observed zero values. However, the quality of the fit needs to be evaluated based upon the intended use of the estimated density. For example, if we were interested in using this estimated model in simulations to understand the spread of the disease in a population with some immunity to the disease, we would want a much better fit to the observed distribution for values near 0 or 1, since the presence of immunity can shorten the incubation period, and in some cases, symptoms will never be demonstrated. In such an instance, it would be imperative that a better model of the behaviour of the incubation period be provided.

Notably, both fitted distributions (MLE and LSE) would be rejected as models based on a goodness-of-fit analysis (see Remark 3). For the maximum likelihood model we find *χ*^2^ = 180.7 (with bin breaks at 0,2,3,4,5,6,*∞* to satisfy *E*_*i*_≥5) and for the least squares model we observe *χ*^2^ = 169.6 (with bin breaks at 0,3,4,5,6,*∞* to satisfy *E*_*i*_≥5). Both of these tests have *p*-values close to zero, and therefore, in both cases, we reject the null hypothesis that the observed data was generated by a log-normal density model. (If the *p*-value is smaller than *α*, then we say that the data are statistically significant at level *α*.)

It should be noted that we chose the log-normal to reflect the choice in [5]. We also considered other distributions, such as the Weibull, which improved the fit of the model (results not shown). Note, however, the Weibull model did not handle the important data near zero well either.

## Measuring the centre of a distribution

To summarize the properties of a given model, such as a density, one often turns to summary statistics, such as the mean, median, or mode. For models such as the normal density, all three of these are equal. However, this is not the case for all density distributions: If the density is skewed, these quantities can be very different. For example, in a right-skewed distribution, the mean is larger than the median.

In [5], a stochastic model was built and employed to simulate influenza dynamics to gain understanding of virus behaviour. The authors then compared the duration of symptoms from the stochastic model with the observed duration: “our estimate of the duration of symptoms (median 7 days) is longer than our model-based estimate (mean 3.4 days)” ([5], page 134). The observed duration of symptoms is right-skewed ([5], Figure four), and therefore the mean will be much higher than the median. Hence, the comparison given above between the observed and model-based estimates of the duration of symptoms is “underplayed” in that the difference between the model-based and empirical data means or medians would be even greater.

## Sample size and hypothesis testing

In biomedical studies, it is generally laborious and expensive to obtain large amounts of quality data, thus, small sample sizes (*n*) are frequently observed. It is therefore important to understand the limitations of the conclusions which can be drawn from such data. In particular, for hypothesis testing, the sample size has a large effect on the power of the statistical procedure (Remarks 4 and 5). Statistical power measures the ability of a test to *correctly* detect the alternative hypothesis. Therefore, conclusions drawn from a small sample population may not be informative.

In [1], the authors compare different measures of immune complex-mediated disease in 2009 H1N1 influenza infection between infants, middle-aged adults, and the elderly ([1], Figures four (a) and four (c)). Here, the sample sizes range from *n* = 3 to *n* = 16 for all groups. In such cases, a more thorough understanding of the difference between the null and alternative hypotheses as well as the inherent variability of the data is important to understand the statistical power of the test statistic being used. Without this additional information, it is difficult to comment on the results. However, the small sample size is an immediate concern.

To gain an understanding of the potential issue, consider the following heuristic example. Suppose that we wish to test if the proportion of the population *p* with a certain characteristic is equal to zero (that is, the null hypothesis is *H*_0_:*p* = 0 and the alternative hypothesis is *H*_*A*_:*p*>0). We collect a sample size of *n* = 10, and observe no one with the characteristic in question. The power of our test depends on what nonzero population proportion we are actually interested in detecting.

Suppose that, if the characteristic were present, it would be present in a large proportion of the population: e.g. *p* = 0.5. In this case, we could be fairly certain that we have an ability to tell the difference between the null (*H*_0_) and alternative (*H*_*A*_) hypotheses: indeed, we would expect to see about half of the sample size with the characteristic (i.e. on average) if *H*_*A*_ holds. On the other hand, suppose that the characteristic of interest is rare in the population: e.g. *p* = 0.05. Here, we would need to sample at least 20 individuals before we could expect to observe at least one individual with the characteristic (again, on average). Thus, with a sample size of *n* = 10, the probability that we could detect a nonzero, but small, population proportion is low, even if the alternative is true. That is, the power of the test to detect *this* population proportion is low.

### Remark 4 (Hypothesis testing)

Once the the null (*H*_0_) and alternative (*H*_*A*_) hypotheses have been set, a decision is made based on the observed data. The decision is either correct or incorrect, and this depends on the data observed and the true state of the world.


We set *α* = *P*(Type I error) and *β* = *P*(Type II error). The hypothesis test rejects *H*_0_ if the data are unlikely to be observed under the null hypothesis. For a concrete example, suppose that we are using the z-test and our hypotheses are *H*_0_:*μ* = *μ*_0_ vs. *H*_*A*_:*μ*>*μ*_0_. Note that the alternative hypothesis in this case is one-sided. With a significance level of *α* = 0.05, we reject the null hypothesis if the observed test statistic


where *σ* is the population standard deviation, *n* is the sample size, and  is the observed sample mean.

### Remark 5 (Power in a hypothesis test).

**Figure 2:**
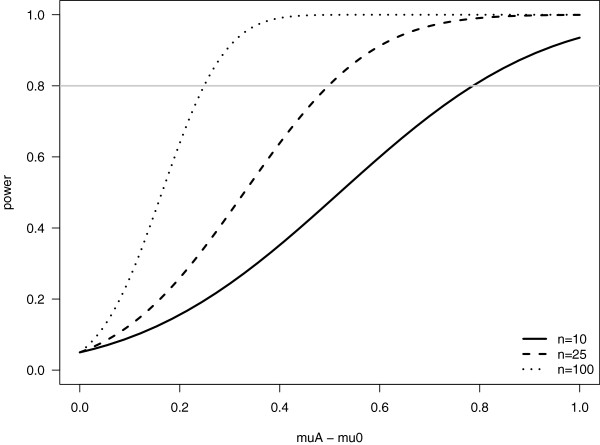
**Power versus effect for various sample sizes.** Three cases are shown for different values of *n* (see legend).

Ideally, the probabilities of both the Type I and Type II errors (defined in Remarks 4) would be small. However, the opposite is true: Decreasing one increases the other. Therefore, the typical approach in hypothesis testing is to fix the probability of a Type I error, *α*, and then to control the probability of a Type II error *β* through the sample size. The power of a hypothesis test is the probability with which we detect the alternative hypothesis, assuming that it is the true state of the world. Hence, the power is equal to 1 - *β*, and we would want this to be high. Exact calculations of power depend on both the test statistic and the actual distribution in the alternative hypothesis. The power of the z-test described in the previous box is the probability that


under the alternative hypothesis, where  denotes the sample mean, now a random quantity (i.e. prior to being observed, or considered under repeated experimentation). However, there are many possible values for the mean in the alternative hypothesis, and we perform the calculations for each population mean *μ*_*A*_∈*H*_*A*_. The value of *μ*_*A*_ used in the calculation should be determined by the experts in the particular field : this is the value of the population mean the scientists would like to detect through the hypothesis test. Overall, the power depends on the difference between the population means under the alternative and null hypotheses (*μ*_*A*_ - *μ*_0_), on how variable the data are (i.e. *σ*), and, perhaps most importantly, on the sample size. Power functions for the one-sided z-test of *H*_0_:*μ* = *μ*_0_ vs. *H*_*A*_:*μ*>*μ*_0_ for different sample sizes are plotted below, assuming that *σ* = 1. We can clearly see that the probability of detecting a fixed difference of *μ*_*A*_ - *μ*_0_ increases, sometimes drastically, as the sample size increases (Figure 2).

## Graphical representation of data

**Figure 3:**
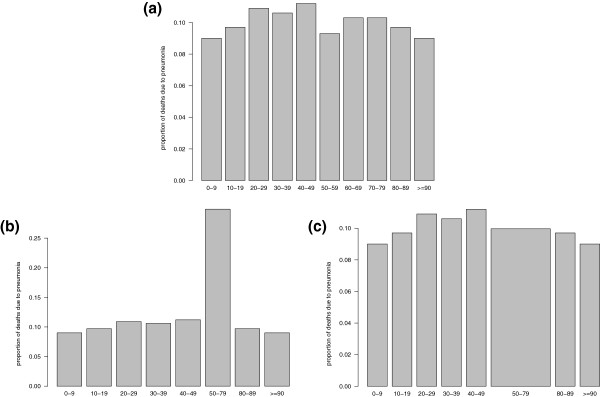
**Three different ways of presenting the same data.** **(a)** shows the original data, while in **(b)** &**(c)** three groups have been amalgamated, however **(c)** reflects the change in bin width in the amalgamated group. Note that Figure 3 **(c)** is a histogram in which the area is correctly proportional to relative frequency while Figure 3 **(b)** is a boxplot which is misleading when the base of the boxes represent different age spreads.

Graphical methods are a simple yet highly effective method of providing information. Popular choices in the influenza literature include histograms or bar plots. The work in [6] studies the 2006–2007, 2007–2008, and 2009 influenza seasons. There, mortality due to severe pneumonia, and morbidity, are compared graphically by age. These figures appear to indicate an increase in the proportion of severe pneumonia deaths and illness in younger adults in 2009 when compared to 2006–2008 (see Figures two and three of [6]). In Figure two of [6], the ages are grouped into bins of equal width (10 years). However, in Figure three of [6], there is a clustered group of width twenty years (25–44) and a clustered group of width ten years (50–59). This results in an apparent spike in the percentage distribution, which can be easily misinterpreted as a significant proportional increase. However, the spike is largely due to the amalgamation of three age groups. A related example for fictional data is illustrated in Figure 3. The figure shows the same data in each panel, but in plot (b) the middle group has been amalgamated in a misleading way. Plot (c), compared to (b), has a lower chance of misinterpretation by the reader.

## Discussion

There is great potential for misrepresentation and misinterpretation within citations and statistical inference. Extra care should be taken so that more clarity is provided when describing study methods and results. Also, more consistent use of statistical methods will provide a clearer picture and enable readers to reproduce results. However, a reader must still consider a paper with a critical mind and approach citation supported inferences and statistical results with a degree of caution. It is important to note that the peer review process is not an ideal mechanism for eliminating errors due to misinterpretation of cited work.

The present work is focused on several specific examples from the H1N1 literature. However, similar challenges are encountered in other subjects and disciplines.

A large number of the misunderstandings presented above could have been resolved with additional information, allowing the reader to examine the data more closely. This point has also been raised in a recent editorial [7], where the question of reproducible research was discussed. In [8] and Strasak et al. [9] have also raised this issue, the latter of which proceeds further, documenting statistical errors common to medical research, some of which include those mentioned here.

## Authors’ information

This research was funded by the Natural Sciences and Engineering Research Council of Canada (JMH, HKJ), Ontario’s Ministry of Research and Education Early Researcher Award (JMH), and York University’s Research at York programme (JR, HKJ, JMH).

## List of notations and abbreviations

MLE: Maximum likelihood estimator
LSE: Least squares estimator
*H*_0_: Null hypothesis
*H*_*A*_: Alternative hypothesis
*μ*: Population mean
*μ*_0_: Population mean under the null hypothesis
*μ*_*A*_: Population mean under the alternative hypothesis
*p*: Population proportion
*σ*: Standard deviation
*α*: Significance level of a test, the probability of a type I error; 1- *α* is equal to the specificity of the test
*β*: Probability of a type II error, 1- *β* is equal to the sensitivity or statistical power of the test
*n*: Sample size.
